# siRNA Targeting Mcl-1 Potentiates the Anticancer Activity of Andrographolide Nanosuspensions via Apoptosis in Breast Cancer Cells

**DOI:** 10.3390/pharmaceutics14061196

**Published:** 2022-06-03

**Authors:** Supusson Pengnam, Purin Charoensuksai, Boon-ek Yingyongnarongkul, Rungnapha Saeeng, Hasan Uludağ, Prasopchai Patrojanasophon, Praneet Opanasopit, Samarwadee Plianwong

**Affiliations:** 1 Pharmaceutical Development of Green Innovations Group (PDGIG), Faculty of Pharmacy, Silpakorn University, Nakhon Pathom 73000, Thailand; patrojanasophon_p@su.ac.th (P.P.); opanasopit_p@su.ac.th (P.O.); 2 Biopharmacy Department and Bioactives from Natural Resources Research Collaboration for Excellence in Pharmaceutical Sciences, Faculty of Pharmacy, Silpakorn University, Nakhon Pathom 73000, Thailand; charoensuksai_p@su.ac.th; 3 Department of Chemistry and Center of Excellence for Innovation in Chemistry, Faculty of Science, Ramkhamhaeng University, Bangkok 10240, Thailand; boonek@ru.ac.th; 4 Department of Chemistry, Faculty of Science, Burapha University, Chonburi 20131, Thailand; rungnaph@buu.ac.th; 5 Department of Chemical and Materials Engineering, University of Alberta, Edmonton, AB T6G 2R3, Canada; hasan.uludag@ualberta.ca; 6 Pharmaceutical Innovations of Natural Products Unit (PhInNat), Faculty of Pharmaceutical Science, Burapha University, Chonburi 20131, Thailand

**Keywords:** siRNA, Mcl-1, andrographolide, synergism, breast cancer, combination index

## Abstract

Breast cancer is the second leading cause of cancer-related death in the US. However, recurrence is frequently found despite adjuvant therapy being available. Combination therapy with cytotoxic drugs and gene therapy is being developed to be a new promising cancer treatment strategy. Introducing substituted dithiocarbamate moieties at the C12 position of andrographolide (3nAG) could improve its anticancer selectivity in the MCF-7 breast cancer cell line. However, its hydrophobicity is one of its main drawbacks. This work successfully prepared 3nAG nanosuspension stabilized with the chitosan derivative NSC (3nAGN-NSC) to increase solubility and pharmacological effectiveness. siRNAs have emerged as a promising therapeutic alternative for interfering with particular mRNA. The 3nAGN-NSC had also induced Mcl-1 mRNA expression in MCF-7 human breast cancer cells at 8, 12, and 24 h. This indicates that, in addition to Mcl-1 silencing by siRNA (siMcl-1) in MCF-7 with substantial Mcl-1 reliance, rationally devised combination treatment may cause the death of cancer cells in breast cancer. The Fa-CI analysis showed that the combination of 3nAGN-NSC and siMcl-1 had a synergistic effect with a combination index (CI) value of 0.75 (CI < 1 indicating synergistic effects) at the fractional inhibition of Fa 0.7. The synergistic effect was validated by flow cytometry, with the induction of apoptosis as the mechanism of reduced cell viability. Our findings suggested the rational use of 3nAGN-NSC in combination with siMcl-1 to kill breast cancer cells.

## 1. Introduction

Breast cancer is a severe health issue that continues to be the focus of scientific research. After lung cancer, it is the second greatest cause of cancer-related fatalities in the United States [1]. Breast cancer may kill about 508,000 people worldwide each year due to its invasiveness and aggressiveness [2]. Breast cancer is a heterogeneous disease, for which treatment strategies depend on its molecular subtypes; for example, endocrine therapy is used for hormone/estrogen-receptor-positive cancer; HER2 targeted therapy is used for human epidermal growth factor receptor 2-positive cancer; chemotherapy; immunotherapy is used for triple-negative breast cancer patients; PARP inhibitors are used for BRCA-mutated triple-negative breast cancer patients [3]. The information on the molecular biology and gene expression markers of breast cancer is crucial for developing new strategies for the disease’s prevention and therapy [3,4]. The overexpression of breast cancer resistance protein, alteration of cell cycle checkpoints, suppression of apoptosis, and activation of various signaling pathways are some of the processes linked to the resistance or relapse of breast cancer [5]. Interestingly, for the anti-apoptosis proteins, a genetic alteration of Mcl-1, involving its amplification, is more common in breast cancer development than Bcl-2 and Bcl-xL amplification in clinical breast cancer datasets and is linked to a worse outcome [6,7]. Due to significant problems, medication resistance and dose-limiting toxicities may also impede patients’ therapeutic efficacy and prognosis [8]. As a result, developing new treatment strategies and more effective agents may be the option to overcome this deadly disease [9]. Therefore, diverse strategies should be utilized concurrently with the synthesis and creation of new treatment modalities [10].

siRNA has particular attention as a possible treatment strategy for genetically related diseases, including malignancies [11,12]. In recent years, potential siRNA has been included in preclinical and clinical investigations for cancer treatment. Atu027 is the delivery vehicle used, containing siRNAs against PKN3, which is the downstream effector of the phosphoinositide-3-kinase signaling pathway PKN3, in order to decrease tumor development and lymph node metastases to solid tumors and is now being explored in a clinical trial [13]. Mcl-1 overexpression resulted in treatment resistance in solid tumors to a variety of chemotherapeutic drugs. Mcl-1 is an anti-apoptotic protein in the BCL-2 family. Mcl-1 inhibits the pore formation of the outer mitochondrial membrane by interacting with the pore-forming effectors (BAK and BAX), resulting in the impeding of the release of cytochrome C and lacking intrinsic apoptosis [14]. Mcl-1 also impacts the cell cycle by changing the regulation of the transcriptional process, mRNA degradation, RNA splicing control, and posttranslational process. Several proteins can bind to Mcl-1 and regulate cell division through mitoses such as the p18 protein, proliferating cell nuclear antigen, and CDK1 [15].

Some studies have found that inhibiting Mcl-1 gene expression can enhance anticancer activity by increasing the levels of apoptotic cascades. Mcl-1 is found in all types of breast cancer. Because recurrent Mcl-1 mutations are rare, cancer cells must find other ways to overcome this susceptibility in order to maintain Mcl-1 expression. Mcl-1 has an important role in tumor formation; most cancer cells, therefore, need to maintain Mcl-1 expression and its stability [16]. MCF-7 cells and Mcl-1-dependent leukemic cells are more vulnerable to chemotherapy than Bcl-2-dependent cells due to their short half-life [17]. When compared to Bcl-2-dependent cells, Mcl-1-dependent leukemic cells are more responsive to chemotherapy. [7]. This suggests that, in tumor cells with high Mcl-1 dependency, a rationally devised combination treatment targeting Mcl-1, as well as single-agent Mcl-1 inhibitors, may trigger cell death in breast cancer [7]. The use of Mcl-1 inhibitors in combination with other critical molecular inhibitors has recently been shown to be a potential method for triggering cell death pathways in cancer therapy [18]. In this work, siRNA (short double-stranded RNA segments) was employed to target Mcl-1 mRNA for initiating the degradation of the Mcl-1 mRNA. The downregulation of Mcl-1 by siRNA has positive therapeutic benefits in breast cancer treatment since it reduces chemotherapy resistance and re-sensitizes tumors to cytotoxic drugs [19].

Natural products have been the subject of scientific research in recent years owing to their favorable effects on human health [20]. Andrographolide (AG) is a natural diterpenoid lactone isolated and discovered from *Andrographis paniculata* [21]. This plant has been used as a traditional herbal medicine for thousands of years in various Asian nations [22]. AG has been linked to various biological actions, including anti-inflammatory and anti-cancer effects. AG has been proven to have considerable potential in cancer treatment in numerous researches, and it caused a low cytotoxicity in normal human breast epithelial cells (MCF-10A) at the tested concentrations [23]. Analogs of AG were shown to cause MCF-7 breast cancer cells to undergo apoptosis [24]. However, it has been discovered that 14-deoxy-11, 12-didehydroandrographolide increases the expression of anti-apoptotic Mcl-1 mRNA by 2.29-fold in the T-47D (human breast carcinoma) cell line [25]. A novel C-12 dithiocarbamate andrographolide (3nAG) analogue has recently been developed and synthesized from natural AG obtained from *Andrographis paniculata*, which is a common Thai herbal plant. Increasing the lipophilicity of AG by adding substituted dithiocarbamate moieties at the C12 position could improve its cytotoxic activity and selectivity on MCF-7 breast cancer cells [26]. However, the primary limitations of its effectiveness are its low water solubility and strong hydrophobicity [27]. Nanosuspensions are now one of the most widely used nanoscale drug delivery technologies [28]. Normally, drug nanoparticles with sizes ranging from 10 to 1000 nm are stabilized by appropriate polymers or surfactants, which have sparked considerable interest in the pharmaceutical sciences, in particular, regarding the passive targeting of chemotherapeutic medicines to cancer cells [29]. Stabilizing nanoparticles by steric and/or electrostatic repulsion are an important strategy. Stabilizers are adsorbed onto the surface of drug nanoparticles in nanosuspension formulations, establishing suitable barriers to avoid aggregation during manufacturing and storage [30].

The formulation of the 3nAG nanosuspensions, on the other hand, has yet to be determined. Because of the thermodynamic instability associated with the high surface free energy of the nanoparticles, a nanosuspension would tend to encounter particle growth and aggregation via the Ostwald ripening phenomena [31]. As a result, finding an appropriate stabilizer in nanosuspension formulations was regarded as crucial. Furthermore, it was considered necessary to find an optimal stabilizer in nanosuspension formulations to overcome low solubility and boost the therapeutic effectiveness of 3nAG. Therefore, we aimed to solve the poor solubility and improve the efficacy of 3nAG by using chitosan derivatives as stabilizers in 3nAG nanosuspensions prepared by a single-step nanoprecipitation (bottom-up) technique. Moreover, this research investigated how 3nAGN-NSC affected Mcl-1 expression and if suppressing Mcl-1 mRNA by siRNA to a particular target makes MCF-7 breast cancer cells more susceptible to 3nAG therapy via the apoptotic pathway induction. For the examination of the synergistic action and dose prediction for 3nAGN-NSC and Mcl-1 siRNA (siMcl-1) targeting apoptosis in the combination treatment, CompuSyn, a computer modeling program, was employed.

## 2. Results

### 2.1. 3nAG Nanosuspension Preparation

**Figure 1:**
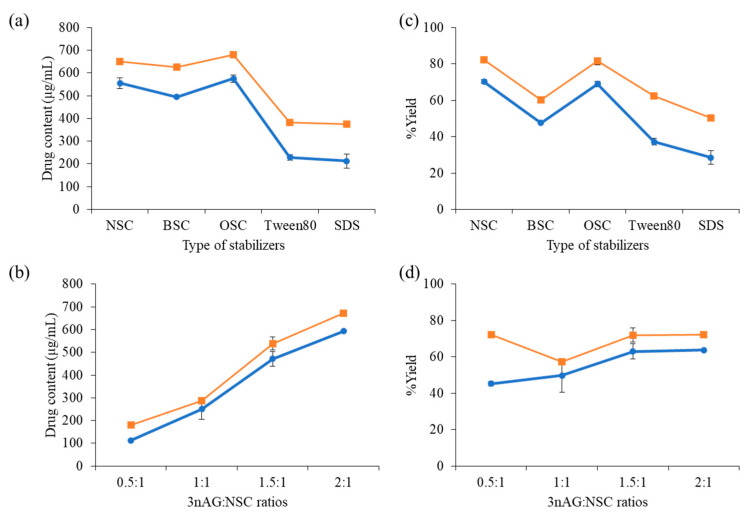
Effects of (**a**,**c**) stabilizer types, and (**b**,**d**) drug:stabilizer ratios (*w*/*w*) on 3nAGN drug content (**a**,**b**) and percentage yield (**c**,**d**) of 3nAGN with (●) or without (◼) sterilization by autoclave. Data are presented as the mean ± standard deviation (n = 3).

The primary issue with nanosuspensions is aggregation or crystal formation. This problem arises as a result of insufficient amounts of stabilizers or the use of unsuitable stabilizer types. To avoid this unfavorable situation, the effects of the type and the amount of stabilizer on the physicochemical parameters of 3nAG nanosuspensions (3nAGN), such as the particle size, zeta potential, and drug content, were examined. The 3nAGN was primarily prepared using chitosan derivatives (naphthyl-grafted succinyl chitosan (NSC), octyl-grafted succinyl chitosan (OSC), and benzyl-grafted succinyl chitosan (BSC)) as a stabilizer at a constant weight ratio of the drug to the polymer of 1.5:1, and this was compared with formulations using surfactants (Tween80 and SDS) as the stabilizer. The formulation prepared using the anti-solvent precipitation technique without the presence of a stabilizer generated a coarse 3nAG suspension which was completely insoluble in water and precipitated at the bottom of the bottle. The findings suggested that 3nAG required a stabilizer to generate nanosuspensions in an aqueous solution. The particle sizes of 3nAG stabilized by chitosan derivatives were around 220–270 nm with a narrow size distribution (PDI 0.07–0.10), whereas those stabilized by both commercial surfactants showed the particle size to be around 860 nm with the PDI of 0.28–0.49 (Table 1). These chitosan derivatives played a critical role in decreasing the drug particles to the nanoscale size. The smallest particle size and the narrowest size distribution were obtained when chitosan derivatives were used as a stabilizer. The zeta potential of all preparations presented a negative value that could provide electrostatic repulsion in order to prevent particle agglomeration. Large particles that were not suspended in the vehicle were eliminated by centrifugation before the drug content of all nanosuspensions was measured by HPLC to calculate the percentage yield (Figure 1). The 3nAGN formulations prepared using commercial surfactants as the stabilizer had a lower drug content and percentage yield when compared with those made using the chitosan derivatives (Figure 1a). The results showed that chitosan derivatives could provide a better stabilization capability than the commercial surfactants Tween80 and SDS. Among these chitosan derivatives, the nanosuspensions formulated using NSC and OSC showed a superior percentage yield when compared with those using BS, so that NSC and OSC were better stabilizers for the preparation of nanosized 3nAG.

The optimal drug:polymer ratios were investigated by increasing the ratio from 0.5 to 2 to identify the ratio that the drug molecule can be physically compatible and completely covered with the stabilizer. The particle size and zeta potential of all formulations were nearly the same, while the ratio of 1:1, 1.5:1, and 2:1 presented a narrow distribution (Table 2). The morphology of the 3nAGN-NSC is depicted in Figure S1. In this study, the NSC was selected for the stabilization of 3nAG, which was coded as 3nAGN-NSC. When the drug-to-polymer ratio was increased, the 3nAG concentration increased (Figure 1b), and the drug-to-polymer ratios appeared to be directly correlated with the drug content. The percentage yield did not change as the ratio increased. To explore the effect of sterilization, 3nAGN was sterilized by an autoclave before examining the anticancer activity. Thus, to ensure the stability of the nanosuspension after its contact with a high temperature (121 °C, 15 psi for 15 min), the drug content and the percentage yield were measured. The results revealed that chitosan derivatives could protect 3nAG from degradation better than the commercial stabilizers (Figure 1a). Sterilization affected the percentage yield of the nanosuspensions, especially at the lower ratio of drug to polymer (0.5:1), which exhibited a significant reduction in the percentage yield (27.11% reduction). At the higher ratios (1:1, 1.5:1, and 2:1), the reductions in the percentage yield were 7.60, 9.05, and 8.49%, respectively (Figure 1b). The 3nAG:NSC ratios of 1.5:1 and 2:1 were optimal for 3nAGN preparation, providing a good percentage yield even after sterilizing with an autoclave. Therefore, 3nAGN-NSC prepared at the 3nAG:NSC ratio of 1.5:1 was selected for further experiments.

### 2.2. Cytotoxicity of 3nAG, 3nAGN-NSC, and siMCl-1

**Figure 2:**
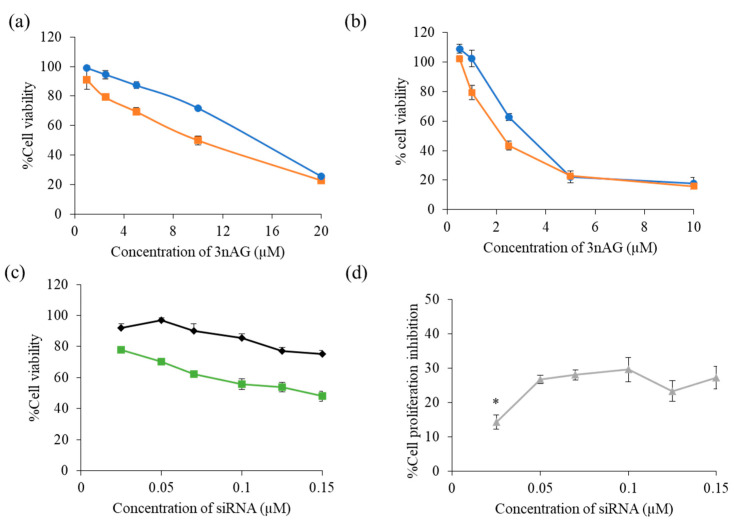
Cell viability of 3nAGN-NSC (◼) and 3nAG suspensions (●) in (**a**) MCF-7 cells, (**b**) MDA-MB-231; (**c**) cell viability of siMcl-1 nioplexes (◼) compared with siNT nioplexes (◆) and (**d**) cell proliferation inhibition of siMcl-1 nioplexes in MCF-7 cells. * The data was significantly different at a *p*-value < 0.05.

The MTT assay was used to evaluate the vitality of the breast cancer cell lines after they were exposed to the 3nAG suspension (as control) or 3nAG:NSC at the weight ratio of 1.5:1 (3nAGN-NSC) in MCF-7 and MDA-MB-231 cells, the results of which are presented in Figure 2a,b, respectively. The same approach was applied to investigate the cytotoxicity of chitosan derivatives only. After a 72 h incubation period with an equivalent dosage of chitosan derivative utilized in nanosuspension formulations, the cell viability of all chitosan derivatives was more than 80%, which indicated a slight effect on cell proliferation (data not shown). The finding referred to the chitosan derivative can be considered a safe stabilizer for nanosuspension. When comparing the IC50 of 3nAGN-NSC to 3nAGN, it decreased from 14.32 ± 0.35 µM (3nAGN) to 11.06 ± 0.42 µM (3nAGN-NSC) in MCF-7 cells and 3.47 ± 0.17 to 2.22 ± 0.14 in MDA-MB-231 cells. The result suggested that the small dimension and wide surface area of the nanoparticles might nonspecifically adsorb on the cell surface and internalize into tumor cells via the endocytosis pathway [32]. This might be explained by the fact that the 3nAG nanosuspension has greater cytotoxicity than the 3nAG suspension. Consequently, the 3nAGN-NSC demonstrated a more significant anticancer effect on the MCF-7 cells. The cell viability of the normal cells was also investigated after treatment with a single treatment of 3nAGN-NSC or siMcl-1, as well as the combination of 3nAGN-NSC:siMcl-1 (Figure S4). The siMcl-1 did not affect the cell viability in normal cells, while 3nAGN-NSC and the combination treatments depicted a slight cytotoxicity to normal cells. At the same concentration, the effect of 3nAGN-NSC and the combination in normal cells exhibited a lower cytotoxicity when compared to breast cancer cells (MCF-7, BT-474, and MDA-MB231 cells). The result revealed that the breast cancer cells are more susceptible to 3nAGN-NSC and combination treatment than the normal cells, which was similar to the cytotoxicity of the previously described AG in normal human breast epithelial cells (MCF-10A) when compared to breast cancer cells (MDA-MB-231, MCF-7, T-47D) at the same concentration [23,33].

The particle size and zeta potential of cationic niosomes were examined prior to using them for siRNA delivery (siNT and siMcl-1), which were 150.50 ± 5.52 nm, and +39.10 ± 1.89 mV, respectively (Figure S3). The concentration of siRNAs targeting Mcl-1 was raised from 0.025, 0.05, 0.07, and 0.1 µM to assess the killing effect of siMcl-1 nioplexes on MCF-7 cells, and the results are displayed in Figure 2b. The growth inhibition was obtained by comparing the effect of siMcl-1 with mock controls (using siNT nioplexes). It was discovered that the increment of the percentage of cell proliferation inhibition of siMcl-1 was not affected by increasing the concentration from 0.05 to 0.15 µM in MCF-7 cells (Figure 2c). The Mcl-1 treatment apparently reduced MCF-7 survival by roughly 26.71–29.64 by the concentration range examined (0.05–0.1) when compared to mock controls (Figure 2c), indicating that MCF-7 cells were particularly vulnerable to Mcl-1 downregulation. As a result, Mcl-1 silencing was pursued further under the hypothesis that combining siMcl-1 with 3nAGN-NSC might minimize the amount of each chemical needed while increasing the inhibitory impact on MCF-7 cell proliferation.

### 2.3. Antitumor Interaction of Combination Treatment

**Figure 3:**
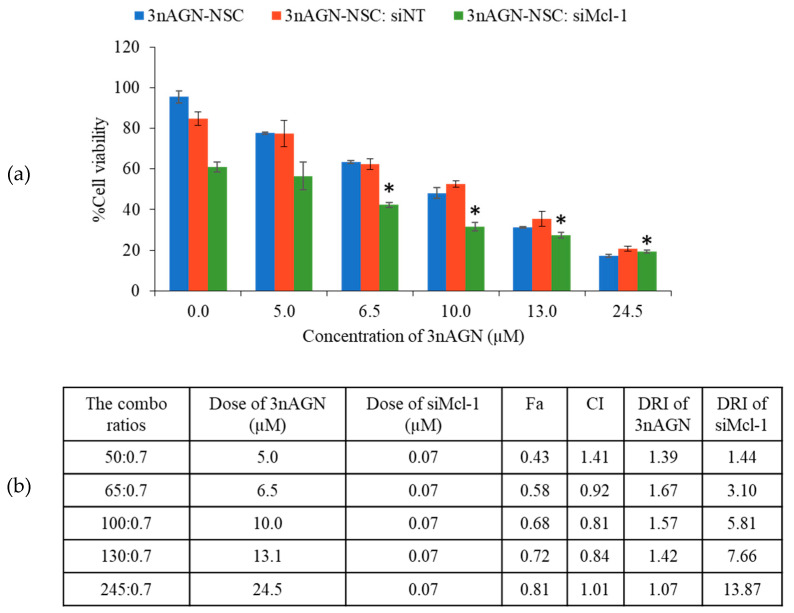
Screening possible synergistic ratio between 3nAGN-NSC and siMcl-1 using non-constant ratios; (**a**) the percentage of cell viability in MCF-7 cells and (**b**) CI values at various ratios of the combination. * The data signify a significant difference from a single treatment with siMcl-1 (*p* < 0.05).

**Figure 4:**
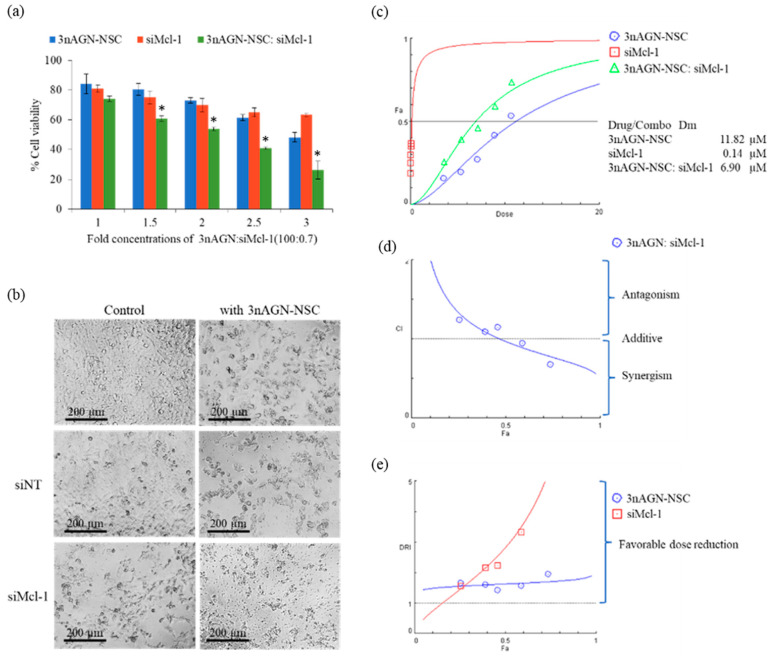
The constant ratio experiment of 3nAGN-NSC:siMcl-1 at 100:0.7; (**a**) the percentage of cell viability in MCF-7 cells and (**b**) morphology of the cells under an inverted fluorescence microscope (40X) after 72-h treatment with 3nAGN-NSC and siMcl-1 at the doses of 10.71 and 0.075 µM, respectively. The actual points are shown as symbols, and the simulated lines were generated from CompuSyn which are presented in (**c**) median effect plots, (**d**) CI plots (CI-Fa), and (**e**) DRI plots (DRI-Fa). * The data signify a significant difference from a single treatment with siMcl-1 or 3nAGN-NSC (*p* < 0.05).

Before assessing the possible synergistic effect of 3nAGN-NSC and siMcl-1, the Fa was calculated by dividing (100% cell viability) by 100 to grasp the concept of the combination index (CI) and to investigate the synergy. The results were then analyzed to see the interaction between 3nAGN and siMcl-1, which would be categorized as follows: synergistic (CI < 1), additive (CI = 1), or antagonistic (CI > 1). Firstly, the effect of combination therapy between 3nAGN and siMcl-1 was primarily screened by using a constant concentration of siMcl-1 at 0.07 µM and different concentrations of 3nAGN-NSC (Figure 3a). Therefore, the ratio of 3nAGN-NSC:siMcl-1 was raised according to the 3nAGN-NSC concentration. The concentration of a single treatment was performed along with the combination. As a result, the synergistic effect was at the ratios of 65–130:0.7, which revealed a CI of less than one (Figure 3a). Furthermore, the dose reduction index (DRI) represents the number of dose reduction folds used in combination when compared to the dose of each drug alone, giving a certain degree of Fa. The data in Figure 3b present that all combinations favored the lowering of the amounts of both drugs by different degrees. The ratio 100:0.7 of 3nAGN-NSC:siMcl-1 was applied for the following tests, demonstrating substantial synergistic activity between 3nAGN-NSC and siMcl-1 with a CI = 0.81 at Fa = 0.68. The relationship between Fa and the dose was verified using CompuSyn software with a constant combination ratio of 100:0.7 to assess the computerized modeling of dose–effect curves, Median effect plots, CI plots (CI-Fa), and DRI plots (DRI-Fa) are presented in Figure 4c–e. The five concentration points of the combination ratio of 100:0.7 with the starting dose of 3nAGN-NSC and siMcl-1 being 3.57 and 0.025 µM, respectively, were performed to observe the synergistic effect along with the designed dose (Figure 4a). The simulated data (line) and actual points (dot) in the dose–effect curves demonstrated the concentration-dependent effect of the combinational treatments. Additionally, the curve of CI plots has an exponential decline trend indicating significant synergistic behavior in MCF-7 at 72 h. Our findings suggested that 3nAGN-NSC and siMcl-1 had a synergistic activity at Fa = 0.50–0.90, with CI values of 0.93–0.58 (Figure 4d), for which the degree of synergy strongly increased as Fa rose.

The possibility of complexation between the 3nAGN-NSC and siMcl-1 nioplexes was investigated by flow cytometry (Figure S2). The results showed that the internalization of siMcl-1 could slightly interfere with the 3nAGN-NSC treatment. The cellular uptake of the combination decreased by 5% from that of siMcl-1 nioplexes (96.35 to 91.39%). It can thus be concluded that the synergistic effect was not caused by the interaction between 3nAG-NSC and siMcl-1 nioplexes before being taken up. The Dm derived from the median effect plots revealed the drug potency (IC50), which was 6.90 µM for the combination treatments (6.85 µM for 3nAGN-NSC and 0.05 for siMcl-1), whereas those for 3nAGN-NSC and siMcl-1 were 11.82 and 0.14 µM, respectively. The doses of 3nAGN-NSC and siMcl-1 were reduced by 1.72- and 2.82-fold, respectively, compared to a single compound dosage (Figure 4c). The data were applied to estimate the dosages of single and combination therapies for reaching the desired Fa. In agreement with previous data, the dosage required to achieve the strong inhibitory effect (Fa >0.7) is more relevant to the therapy than the low effect [34]. Based on the Fa of 0.7 from the 3nAGN-NSC and siMcl-1 treatments, the combination demonstrated a favorable dose reduction and a substantial synergism of 3nAGN-NSC and siMcl-1 at 1.84 and 4.72, respectively, which provided CI at 0.75. Therefore, an Fa of 0.7 was chosen for further studies. The cell density and morphology of the treated cells are presented in Figure 4b. It was observed that when compared to cells treated separately with each drug at the same dose, the density of cells treated with the combination was much lower and the prevalence of shrunk and detached cells increased. In the previous study, the effect of Mcl-1 downregulation significantly declined the cell viability of MCF-7 cells, while the viability of MDA-MB-231 was not altered [35]. The effect of combination treatment was also investigated in BT-474 and MDA-MB 231 cells (Figure S5b,c). The results revealed that the combination of 3nAGN-NSC and siMcl-1 yielded cytotoxicity in BT-474 cells but did not enhance the cytotoxicity to MDA-MB-231 cells when compared with a single treatment.

### 2.4. Mcl-1 Expression of Andrographolide Treatment

**Figure 5:**
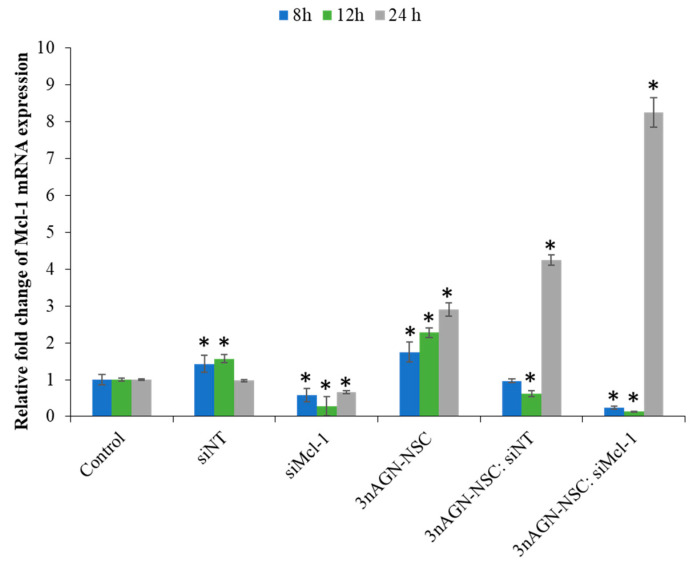
The mRNA expression study was examined using the △△CT method, which is presented as a percentage of the relative fold change of Mcl-1 mRNA expression in the MCF-7 cells. * The data are significantly different from control in each time point (*p* < 0.05).

Mcl-1 mRNA expression was increased in T-47D breast cells after a 24 h treatment with 14-deoxy-11,12-didehydroandrographolide, according to previous research [25]. After treatment with 10.71 µM 3nAGN-NSC at various time intervals, our results showed that Mcl-1 mRNA levels were increased depending on the incubation times at 8, 12, and 24 h. An RNA interference technique was used to decrease Mcl-1 expression in MCF-7 cells. At the siMcl-1 concentration of 0.075 µM, the qPCR showed the efficient and consistent downregulation of Mcl-1 gene expression in the absence of 3nAGN-NSC (Figure 5). When compared to the untreated control, the transfection led to a substantial reduction in Mcl-1 transcript levels: 0.58 ± 0.16, 0.28 ± 0.26, and 0.65 ± 0.04 at 8, 12, and 24 h, respectively. For the 3nAGN-NSC and siMcl-1 combination treatment group, the fold change of Mcl-1 mRNA expression was significantly lowered by 0.24 ± 0.04 and 0.13 ± 0.01 at 8 and 12 h, respectively. The combinational group with 3nAGN-NSC:siMcl-1 (10.71 and 0.075 µM) had a greater drop in Mcl-1 mRNA than the untreated and single-drug groups at 8 and 12 h of treatment. At 24 h, however, the Mcl-1 transcript levels of the combination treatment showed a marked increase to 8.25 ± 0.40. These findings suggested that Mcl-1 silencing may activate apoptotic signaling molecules. This might explain its synergistic action, which could be used as part of a prospective combination strategy involving medications that target Mcl-1 with andrographolide.

### 2.5. Apoptosis Induction

**Figure 6:**
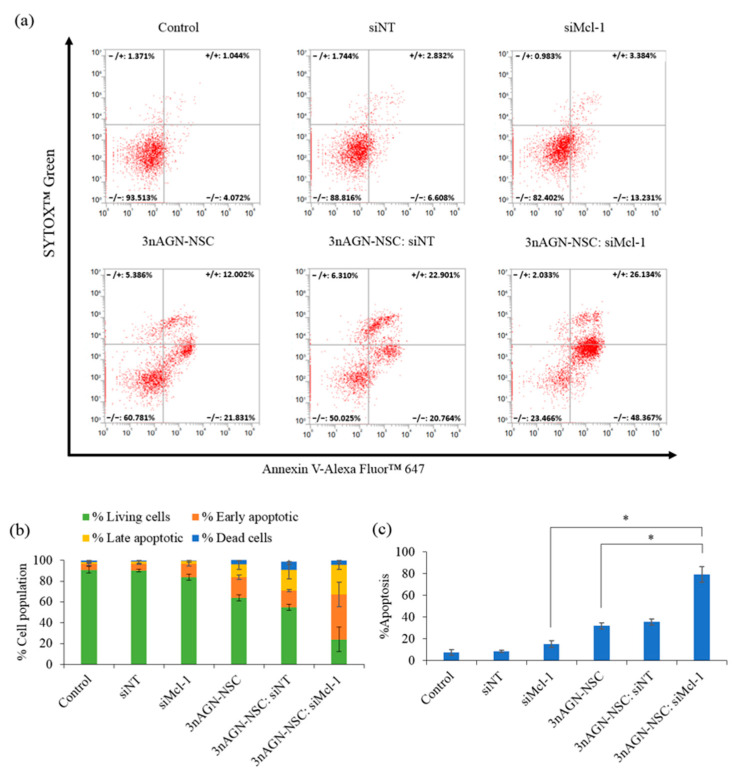
Apoptosis profile of MCF-7 cells analyzed by a flow cytometer. The cell was treated with 3nAGN (10.71 µM), siMcl-1 (0.075 µM), siNT (0.075 µM), 3nAGN-NSC:siNT (10.71 and 0.075 µM), or 3nAGN-NSC:siMcl-1 (10.71 and 0.075 µM) for 24 h and stained with Annexin V-Alexa Fluor^™^ 647/SYTOX^™^ Green. (**a**) Representative scatter plots of SYTOX™ Green (y-axis) vs. annexin V (x-axis). (**b**) Percentage of living, early apoptotic, late apoptotic, and dead cells. (**c**) the percentage of apoptosis. * The data are significantly different (*p* < 0.05).

MCF-7 cell apoptosis was measured using flow cytometry to observe the effect of the combination treatment. The MCF-7 cells were treated either by PBS (control), 3nAGN-NSC (10.71 µM), siMcl-1 (0.075 µM), siNT (0.075 µM), 3nAGN-NSC:siNT (10.71 and 0.075 µM), or 3nAGN-NSC:siMcl-1 (10.71 and 0.075 µM) in combination at a simulated concentration that provided an Fa of 0.7, as shown in Figure 6. The value of synergism in the apoptotic pathway was proven by adjusting the quadrants to correlate with the death of controls. Alexa Fluor™ 647 coupled with Annexin V was used to measure the apoptotic cells, whereas SYTOX™ Green was used to stain the necrotic or dead cells. In a single treatment or combination with 3nAGN-NSC, the untreated cell and mock control (siNT) showed no significant cell death. Apoptosis was determined to be the cell death mechanism of 3nAGN-NSC (32.05 ± 2.87%), which was observed by early apoptosis and late apoptosis at 19.94 ± 2.24 and 12.12 ± 0.98%, respectively. This is in line with the findings of the siMcl-1 treatment, which showed that apoptosis (15.37 ± 2.92%) was the dominating process. However, when compared to the combination treatment, a single therapy showed a lower proportion of early and late apoptosis. The cell apoptosis rate of the combination was 79.50 ± 7.07%, which consisted of early and late apoptosis at 43.36 ± 11.72 and 28.53 ± 3.66%, respectively.

**Figure 7:**
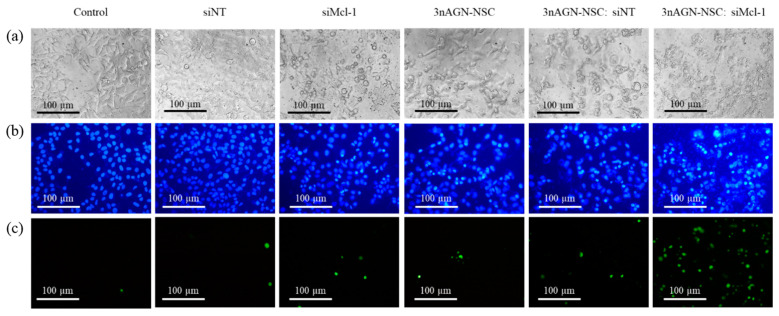
Double-stain apoptosis detection was observed under an inverted fluorescence microscope (100X) after incubation with 3nAGN (10.71 µM), siMcl-1 (0.075 µM), siNT (0.075 µM), 3nAGN-NSC:siNT (10.71 and 0.075 µM), or 3nAGN-NSC:siMcl-1 (10.71 and 0.075 µM) for 24 h; (**a**) bright field images, (**b**) fluorescence images of Hoechst 33342 staining (blue fluorescent cells), (**c**) fluorescence images of SYTOX™ Green staining (green fluorescent cells).

We used double labeling of apoptotic cells with membrane-permeating Hoechst (blue) and impermeable SYTOX™ Green. The apoptosis death mechanism was confirmed based on nuclear condensation, given by the more intense blue fluorescence than in healthy cells. At the loss of cell integrity, SYTOX™ Green intercalates the DNA base pair, illuminating the green fluorescent color [36]. The morphology of the untreated and siNT-treated cells showed a smooth–spheroid nucleus with a normal chromatin distribution, as demonstrated by the uniform blue fluorescence and a lack of green fluorescent cells, as seen in the fluorescence images (Figure 7). The results showed that the cell density dropped after the treatment with 3nAGN-NSC or siMcl-1, and the cell apoptosis was evident (bright blue fluorescent). In addition, apoptosis caused cell shrinkage, which reduced the nucleus and cytoplasm volume. Furthermore, as compared to single treatments, chromatin condensation increased considerably in the combination treatment, as seen by cells showing the bright blue fluorescence of the apoptotic chromatin. Noticeably, the combination therapy revealed green fluorescence, which might be the result of cell membrane rupture during late apoptosis or necrosis. This was consistent with the flow cytometry analysis of the combination treatment. As a result, the combination therapy appeared to have more potent cytotoxic effects and a greater rate of apoptosis in both the early and late stages than the individual treatments.

## 3. Discussion

The 3nAG was reported to have specific cytotoxicity on MCF-7 cells [26]. Because of its high hydrophobicity from adding substituted dithiocarbamate moieties at the C12 position of AG, 3nAG might be one of the most challenging substances to treat cancer in a clinical context. The anti-solvent approach was used to effectively construct andrographolide analogue (3nAG) nanosuspensions, which were stabilized by amphiphilic chitosan derivatives (NSC, OSC, BSC) and surfactants (Tween80 and SDS). These techniques were successful in improving the water solubility of the hydrophobic drug. The nanometer size range, size distribution, highly negative charge, and high drug content of 3nAG stabilized with NSC at a drug-to-polymer ratio of 1.5:1 (*w*/*w*) (3nAGN-NSC) indicate various benefits. Even after sterilization, there was no significant degradation of 3nAG, indicating that the medication of 3nAG and chitosan derivatives (NSC) was compatible and practical in parenteral use. Surprisingly, the 3nAGN-NSC demonstrated a greater anticancer efficacy than the 3nAG suspension (without stabilizer) against MCF-7. These findings suggested that 3nAGN-NSC is a potential formulation for improving its water solubility and anticancer activity. According to this research, the hydrophobic naphthyl groups adsorbed onto the drug’s surface may interact with the hydrophobic drug via hydrophobic interactions at various force levels. In this experiment, the zeta potential of all 3nAGNs was similar to a negatively charged surface with a zeta potential of at least 30 mV [37,38]. These negatively charged nanoparticles might occur by coating a chitosan derivative onto the 3nAG, which caused repulsion and established an electrostatic force between them. Multiple grafted hydrophobic groups also induced a steric effect on the chitosan polymer, which is known as naphthyl (double aromatic ring) in nanosuspension. The double stabilizer action, which combined electrostatic and steric repelling forces, minimized and prevented particle aggregation [39]. These barriers appear to be in charge of avoiding particle aggregation and ensuring particle stability [31].

The Mcl-1 levels had risen from 1.75–2.90 throughout the study time in a time-dependent manner. Our findings were evaluated at the mRNA level after 3nAGN-NSC treatment for various time intervals (8, 12, and 24 h). Bcl-2 and Mcl-1 are overexpressed in some cancer cells, particularly after treatment [40]. As a result of the decrease of Mcl-1 mRNA, we expect that siMcl-1 will reduce the apoptotic threshold and boost 3nAGN-NSC’s therapeutic efficacy [41,42]. Mcl-1 expression is increased in a variety of malignancies, making cells resistant to chemotherapy-induced apoptosis [43,44]. Because Mcl-1 has a very short half-life (usually referred to as 1 h) when compared to other antiapoptotic proteins, it may be possible to battle these tumors by sensitizing Mcl-1-dependent cancer cells to chemotherapy-induced apoptosis when Mcl-1 is inhibited [45]. The therapeutic effect of combination therapy used as medications, on the other hand, is determined by the regimen, dose or drug ratios, and the mechanism of action of the compounds. Certain ratios and administration regimens are synergistic for some kinds of chemicals, whereas others are antagonistic [46,47]. The significant aspects of combinational treatment are lower dosages and increased chemicals [48,49]. We compared 3nAGN-NSC with siMcl-1 in this investigation, according to a fixed ratio of 3nAGN-NSC:siMcl-1 at 100:0.7. The IC70 (Fa 0.7) value for 3nAGN-NSC as a single dose was 20.29 µM after 72 h of treatment. According to the constant ratio experiments shown here, combining 3nAGN-NSC and siMcl-1 strongly inhibited the growth of MCF-7 cell cultures at lower concentrations of 11.01 and 0.077 µM of 3nAGN-NSC and siMcl-1, respectively, for which the concentration used for a Fa of 0.7 and a CI of 0.75 was 1.84 times lower than 3nAGN-NSC and 4.72 lower than siMcl-1 alone. For anticancer properties with high inhibition effects, especially those in the Fa 0.7–0.9 range, these are more relevant to the therapy than those with a low inhibition effect [47].

Our findings revealed that simulated data of combining 3nAGN-NSC and siMcl-1 to reach a high Fa provided a more synergistic level. The combination of 3nAGN-NSC with siMcl-1 not only provides the synergistic effect but also lowers the dosage concentration. There is no published research on the effects of combining 3nAGN-NSC and siMcl-1 in cancer treatment. However, the mechanism through which 3nAG causes a rise in Mcl-1 is unclear, and further research is required to clarify it. The upregulation in the Mcl-1 level might cause an increase in apoptosis, cell differentiation, and the AKT-mTOR-Bcl-2 signaling pathway after the treatment of AG [25]. As expected, siMcl-1 decreased Mcl-1 expression in the intrinsic apoptotic pathway, which is a crucial signaling molecule in downstream apoptosis. Mechanism investigations have recently revealed the efficacy of andrographolide against breast cancer [50,51]. Andrographolide could induce cell death via several pathways [52,53]. According to the results of the previous study, the anticancer activity of SRJ09, (3,19-(2-bromobenzylidene) andrographolide) was mediated by causing G1-phase cell cycle arrest via the stimulation of p21 expression along with a reduction of CDK-4 expression, which eventually resulted in the onset of apoptotic cell death [24,54]. Interestingly, in MCF-7 breast cancer cells, the mechanism by which SRJ09 reduces cell viability is attributable to the development of an extrinsic apoptotic pathway independent of Bcl-2 and p53 [24,54]. This corresponds well with the findings involving the effects of andrographolide, whereby the treated cells did not show any change in the Bcl-2 expression level, which is a vital protein preventing the release of cytochrome c from mitochondria in the intrinsic apoptosis pathway [55,56]. SRJ09 was believed to trigger cell death via an extrinsic apoptotic mechanism involving signaling by the cell surface death receptor, which is unaffected by Bcl-2 regulation.

The Annexin V, Alexa FluorTM 647 conjugate staining was used to validate the apoptotic cell death mediated by 3nAGN-NSC. The membrane phospholipid phosphatidylserine is externalized to the outer surface of the cell membrane in apoptotic cells, and this process happens at the very early stages of apoptosis. Combination treatment has shown to be the most effective in terms of apoptosis induction. Its advantage in terms of apoptosis induction comes via several different pathways, which may effectively reduce drug resistance because cancer cells are typically incapable of responding to the harmful effects of two therapeutic drugs at the same time [57,58]. In addition, the anticancer activation of pro-survival signaling (Mcl-1) might be compromised if 3nAGN-NSC activates them. The efficacy of 3nAGN-NSC can be greatly improved by targeting the Mcl-1 pro-survival signaling pathway [59,60]. As a result, identifying pro-survival signaling has significant therapeutic implications. Our study provided a new therapeutic strategy for the diverse usage of 3nAG and drugs targeting Mcl-1 downregulation as a combination chemotherapeutic treatment.

## 4. Materials and Methods

### 4.1. Materials

A 12-dithiocarbamate-14-deoxyandrographolide analogue (3nAG) with >99% purity was generously supplied by Dr. Rungnapha Saeeng from Burapha University, Thailand. The plier-like cationic lipid B (PCL-B) was kindly assisted by Dr. Boon-ek Yingyongnarongkul from Ramkhamhaeng University, Thailand. Cholesterol (Chol) was acquired from Carlo Erba Reagent (Cornaredo, MI, Italy). N-naphthyl-N-Osuccinyl chitosan (NSC), N-octyl-N-O-succinyl chitosan (OSC), and N-benzyl-N,O-succinyl chitosan (BSC) were kindly gifted from Dr. Warayuth Sajomsang from National Nanotechnology Center (NANOTEC), Thailand. The dialysis bag (Cellusep^®^ T1 6000–8000 MWCO) was purchased from Membrane Filtration Products (Seguin, TX, USA). Dimethyl sulfoxide (DMSO) was obtained from Merck & Co. (Darmstadt, Germany). Sodium dodecyl sulfate (SDS), Tween80, Span20 and 3-(4,5-dimethyl-thiazole-2-yl)-2,5-diphenyl tetrazolium bromide (MTT) were purchased from Sigma Aldrich^®^ St. Louis (MO, USA). The siRNA-targeting apoptosis-related protein Mcl-1 was obtained from Ambion™ Silencer™ Validated siRNA (Cat# 4390824) Thermo Fisher Scientific (Waltham, MA, USA). BJ-5ta, MCF-7, BT-474, and MDA-MB-231 cell lines were obtained from the American Type Culture Collection (ATCC, Manassas, VA, USA). All culture reagents were purchased from Gibco BRL (Rockville, MD, USA).

### 4.2. 3nAG Nanosuspension Preparation

For the formulations of 3nAG nanosuspensions (3nAGN) presented in Table 1, bottom-up process technology was applied with the solvent-anti-solvent technique [61]. Firstly, the 3nAG and stabilizers were dissolved in 1.5 mL of DMSO as an organic solvent. The weight ratios of the 3nAG and stabilizers (chitosan derivatives: NSC, OSC, BSC, and surfactants: Tween80 and SDS) were varied as 0.5:1, 1:1, 1.5:1, and 2:1. The anti-solvent (3.5 mL of ultrapure water) was prepared in a bottle with constant magnetic stirring before the slow dropping of the organic phase. After stirring for 24 h at 25 °C, the mixture was subsequently put into a dialysis tube (6000–8000 MWCO) and dialyzed against distilled water to remove the organic solvent. 3nAG, stabilized by either chitosan derivatives, SDS, or Tween80, was then centrifuged at 1500 rpm for 5 min, and the supernatant, which contained 3nAGN, was collected for further experiments. The drug solution in DMSO was progressively poured into 3.5 mL of an aqueous solution containing the stabilizers to generate the formulation comprising the commercial stabilizer. All 3nAGN formulations were sterilized in an autoclave before using them in cell culture experiments.

### 4.3. Particle Size and Zeta Potential

The particle size and zeta potential of the formulations were assessed in distilled water with 100-time dilution using a the Zetasizer Nano ZS from Malvern Instrument (Malvern Co., Worcestershire, UK). At 25 degrees Celsius, the liquid sample was measured three times with 12 scans and a scattering angle of 90 degrees. All data are reported as the mean and standard deviation (SD). The morphology of nanosuspensions was analyzed using a transmission electron microscopy (TEM, JEM-2100, JEOL Co., Ltd., Tokyo, Japan). Before the observations, which were performed at an accelerating voltage of 80 kV, the formulations were dried on a formvar–carbon film and stained with 1% uranyl acetate.

### 4.4. Drug Content Analysis by High-Performance Liquid Chromatography (HPLC)

The 3nAG content of 3nAGN was measured using an HPLC (Agilent 1100 series, Agilent Technologies, Santa Clara, CA, USA) after 3nAGN was entirely dissolved in methanol. The apparatus was connected with a reverse-phase Phenomenex-C18 column (4.6150 mm, 5 m) and a UV detector set at 210 nm. The measurement was performed at 25 °C. The elution condition was set with a mobile phase consisting of a 20:80 *v*/*v* mixture of water and methanol, a flow rate of 1.0 mL/min, and an injection volume of 20 mL. A standard drug solution with various concentrations was used to generate a standard curve with an R^2^ value of 0.999. All of the samples were examined three times. The concentration of 3nAG was estimated using a standard curve, and the percentage yield of 3nAG was computed as follows: %yield = An/A0 × 100, where An and A0 signify the quantity of a drug determined from 3nAG and the amount of a drug utilized for preparation, respectively.

### 4.5. In Vitro Cytotoxicity

An MTT assay was used to assess the cytotoxicity of all formulations on MCF-7, BT-474, and MDA-MB-231 human breast cancer cell lines. The breast cancer cells were cultivated in Dulbecco’s Modified Eagle Medium (supplemented with 10% fetal bovine serum and 1% penicillin–streptomycin) and then incubated at 37 °C and 5% CO_2_. A 96-well plate was seeded with the breast cancer cells at 7 × 10^3^ cells per well. After a 24-h incubation period, the samples in the medium were added to the plate and incubated for another 72-h period under normal conditions. A control experiment with untreated cells was also carried out. The cells were then incubated with MTT for 3 h at a final concentration of 1 mg/mL for each well. For the dissolving of the formazan crystal, the medium was replaced with 100 µL of DMSO. A PerkinElmer’s VICTOR Nivo^®^ Multimode Microplate Reader was used to measure the absorbance at 550 nm. The percentage of cell viability was calculated using absorbance at 550 nm and was compared to the control, which was defined as 100% cell viability.

### 4.6. Antitumor Interaction of 3nAGN-NSC and siMcl-1 Delivery

Cationic niosomes containing Span20, Chol, and PCL-B were used as transfection agents in this study [35]. The niosomes were formulated by the thin film hydration method followed by probe sonication. The siMcl-1 or siNT was mixed with transfection agents for 30 min before transfection. The weight ratio of cationic niosomes/siRNA was fixed at 15 (*w*/*w*). An MTT assay was used to investigate the synergistic effect of 3nAGN and siMcl-1 utilizing two complementary approaches, namely non-constant and constant ratios. The concentration of siMcl-1 was kept constant at 0.07 µM for all treatment conditions in the non-constant ratio experiment, whereas the concentrations of 3nAGN-NSC were raised as follows: 5.0, 6.5, 10.0, 13.0, and 24.5 µM.

Cells were treated with 5 different doses of 3nAGN-NSC and siMcl-1 for the constant ratio experiment, whereas the ratio of 3nAGN-NSC:siMcl-1 was kept constant at 100:0.7 for the concentration alteration studies. The fold concentration was raised from 1, 1.5, 2, 2.5, and 3-fold, with the lowest concentration of 3nAGN-NSC and siMcl-1 combination doses being 3.57 and 0.025 µM, respectively, and the maximum concentration being 10.71 and 0.075 µM. Furthermore, for the control, cells were treated with each drug in a single treatment at the same dose as in the combination trials. For all situations, percentage cell viability was acquired and integrated into the CompuSyn software for the computation of synergism.

### 4.7. Analysis by CompuSyn Software

CompuSyn software is an algorithm to determine the combination index (CI) and the dose reduction index (DRI) for all situations, and these values were integrated into the calculation for synergism and the DRI. Percentages of cell viability were converted to fractions of effect (Fa), which were calculated by (100% cell viability) divided by 100. The actual data points, including treatment concentrations (µM) and Fa, were entered into the program to simulate combination data. The CI was defined as CI < 1 (synergist), CI = 1 (additive), and CI > 1 (antagonism). When compared to the concentration of the same drug employed singly to have the same inhibitory effect, the DRI value indicated the fold decrease in the concentration of each agent in combination. The DRI value is defined as DRI = 1 (no dose reduction), DRI > 1 (favorable dose reduction), and DRI < 1 (unfavorable dose reduction).

### 4.8. mRNA Expression Level by Real-Time PCR

Mcl-1 mRNA expression was measured after MCF-7 cells were treated for 24 h. In a 96-well plate, mRNA was extracted from cultivated cells and converted to cDNA using the SuperPrep™ II Cell Lysis & RT Kit for qPCR (Toyobo, Osaka, Japan). The generated cDNA was evaluated by quantitative real-time PCR in a LightCycler^®^ 480 Instrument II (Roche, Basel, Switzerland) with annealing at 59 °C for 30 s, using Thunderbird™ SYBR^®^ qPCR Mix (Toyobo, Japan). The relative mRNA expression was calculated by the delta–delta CT method (△△CT method). Specific primers spanning exons of GAPDH and Mcl-1 were designed from NM_001357943.2 and NM_021960.5, respectively. GAPDH forward primer: TTTTGCGTCGCCAGCCG, GAPDH reverse primer: CGCCCAATACGACCAAATCC (product length 84 bp), Mcl-1 forward primer; GGAGACCTTACGACGGGTT, Mcl-1 reverse primer: AGTTTCCGAAGCATGCCTTG (product length 75 bp).

### 4.9. Apoptosis Evaluation

The cell death pathway of MCF-7 cells following the treatment with a combination of 3nAGN-NSC and siMcl-1 was observed, utilizing a double staining approach and examined under an inverted microscope with fluorescence detection, compared to single-compound treatments. First, the cancer cell line was cultured and seeded as 10,000 cells per well on a 96-well plate, then incubated to confluence in a controlled normal environment. After that, the formed samples including PBS (control), 3nAGN-NSC (10.71 µM), siMcl-1 (0.075 µM), siNT (0.075 µM), 3nAGN-NSC:siNT (10.71 and 0.075 µM), or 3nAGN-NSC:siMcl-1 (10.71 and 0.075 µM) were introduced to the cells with concentrations providing Fa 0.7 and were incubated for another 24 h. The cells were then stained for 15–30 min with 10 µg/mL Hoechst 33342 (10 µL) and 0.5 µM SYTOX™ green nucleic acid stain (5 µL) before being observed under an inverted fluorescence microscope to determine the cell-killing process.

Flow cytometry was used to further investigate the apoptotic induction in the treated cells. MCF-7 cells were seeded at a density of 3 × 104 cells per well in 48-well plates and incubated overnight. The cells were then incubated for 24 h with PBS (control), 3nAGN-NSC (10.71 µM), siMcl-1 (0.075 µM), siNT (0.075 µM), 3nAGN-NSC:siNT (10.71 and 0.075 µM), or 3nAGN-NSC:siMcl-1 (10.71 and 0.075 µM). The cell samples were collected at the designated time, and the cells were washed twice with DMEM without serum before being trypsinized with 0.05% trypsin–EDTA. The cells were then centrifuged for 5 min at 1000 rpm and rinsed three times with PBS. After being suspended in 100 µL 1×Annexin V Binding Solution, the cells were stained with Annexin V, Alexa Fluor™ 647 conjugate (5 µL), and 0.5 µM SYTOX™ green (5 µL) for 15 min in the dark. Before analyzing the sample with a flow cytometer, the binding buffer (400 µL) was added to the sample.

### 4.10. Statistical Analysis

The investigations were carried out in triplicate, and the numerical data are presented as means and standard deviations (SD). Microsoft^®^ Excel 2019 was used to perform the analysis of variance (ANOVA), followed by an LSD post hoc test with a 95 percent confidence interval; a significant difference was proclaimed at *p* < 0.05.

## 5. Conclusions

This study effectively formulated C-12 dithiocarbamate andrographolide analogue (3nAG) nanosuspensions stabilized by an amphiphilic chitosan derivative (NSC) (3nAGN-NSC) which provided improved anticancer efficacy when compared to 3nAG aqueous suspensions. Furthermore, this research suggested that siMcl-1 was able to reduce 3nAG-induced Mcl-1 overexpression and sensitize 3nAGN-NSC cytotoxicity to MCF-7 human breast cancer cell lines by downregulating Mcl-1 mRNA via the apoptosis induction pathway. The synergistic effect of the combination was interpreted using CompuSyn software. At the projected 70 percent inhibitory response, the combination allowed dosage reductions of 3nAGN-NSC and siMcl-1 of 1.84 and 4.72, respectively (Fa 0.7, CI 0.75). Finally, this work provided a new therapeutic approach for the diversified use of andrographolide analogues (3nAG) in combination with a compound targeting Mcl-1 downregulation, which might lead to a superior response to single-agent therapy.

## Figures and Tables

**Table 1:** The effect of stabilizer types on the particle size, polydispersity index (PDI), and zeta potential of 3nAGN. The means and standard deviations of triplicate experiments are used to represent the data.

Stabilizers	Size		PDI		Zeta Potential	
	Mean	SD	Mean	SD	Mean	SD
NSC	249.6	1.6	0.075	0.007	−26.6	0.5
BSC	270.5	1.3	0.109	0.019	−26.6	0.9
OSC	222.2	3.2	0.088	0.013	−31.7	1.0
Tween80	868.7	18.5	0.282	0.023	−42.8	0.6
SDS	862.1	36.7	0.487	0.018	−29.6	0.4

**Table 2:** The particle size, polydispersity index (PDI), and zeta potential of 3nAGN prepared with different ratios of 3nAG:NSC. All data represent the mean ± standard deviation (n = 3).

3nAG:NSC Ratios	Size		PDI		Zeta Potential	
	Mean	SD	Mean	SD	Mean	SD
0.5:1	239.20	3.82	0.209	0.009	−28.93	1.01
1:1	213.53	2.25	0.062	0.007	−19.17	1.25
1.5:1	222.50	1.49	0.085	0.027	−25.63	0.23
2:1	235.50	0.79	0.052	0.030	−27.30	0.20

## Data Availability

Not applicable.

## Supplementary Materials

The following supporting information can be downloaded at: https://www.mdpi.com/article/10.3390/pharmaceutics14061196/s1, Figure S1: Morphology of (a) 3nAG suspension and (b) 3nAGN-NSC under 100X inverted mi-croscope and (c) TEM image of 3nAGN-NSC, Figure S2: (a) Cellular uptake and (b) mean fluorescent intensity of siAF488 transfected with cationic niosomes and siAF488 in the combination with 3nAGN-NSC in MCF-7 cells, Figure S3: Characerization of cationic niosomes: (a) size distribution (mean particle size of 150.50 ± 5.52 nm) and (b) zeta potential distribution (mean zeta potential of +39.10 ± 1.89 mV) obtained from the Zetasizer Nano ZS, Figure S4: The cell viability of a single treatment with 3nAGN-NSC or siMcl-1 and the combina-tion of 3nAGN-NSC: siMcl-1 (The tested concentration was nearly the IC50 of 3nAGN-NSC in each breast cancer cell) in normal human fibroblast cells (BJ-5ta cells), and breast cancer cells (MCF-7, BT-474 and MDA-MB-231 cells), which was examined by MTT assay. The cell was tested in the same condition with the method Section 4.5. The BJ-5ta cells were maintained in DMEM containing 10% FBS, 1% l-glutamine, 1% non-essential amino acids solution, 100 U/mL penicillin, and 100 mg/mL streptomycin at 80% confluency before seeding. * The data is significantly different from BJ-5ta (*p* < 0.05), Figure S5: The cell viability of (a) MCF-7 with the combination 3nAGN-NSC: siMcl-1 (100: 0.07, 10 and 0.07 µM, respectively), (b) BT-474 after treatment with the combination 3nAGN-NSC: siMcl-1 (100: 0.07, 7.14 and 0.05 µM, respectively) and (**c**) MDA-MB-231 after treatment with the combination 3nAGN-NSC: siMcl-1 (50: 0.07, 3.57 and 0.05 µM, respectively). * The data is significantly different (*p* < 0.05).
