# Supplementary material for: siRNA Targeting Mcl-1 Potentiates the Anticancer Activity of Andrographolide Nanosuspensions via Apoptosis in Breast Cancer Cells
Source: Pharmaceutics. 2022 Jun 3;14(6):1196. doi: 10.3390/pharmaceutics14061196 (PMC9230779; doi:10.3390/pharmaceutics14061196)
Supplement: Supplementary file 1 [file pharmaceutics-14-01196-s001.zip › pharmaceutics-1732774-supplementary.pdf]

pharmaceutics

MDPI

Supplementary Materials: siRNA Targeting Mcl-1 Potentiates the Anticancer Activity of Andrographolide Nanosuspensions via Apoptosis in Breast Cancer cells

Supusson Pengnam, Purin Charoensuksai, Boon-ek Yingyongnarongkul, Rungnapha Saeeng, Hasan Uludağ, Prasopchai Patrojanasophon, Praneet Opanasopit and Samarwadee Plianwong

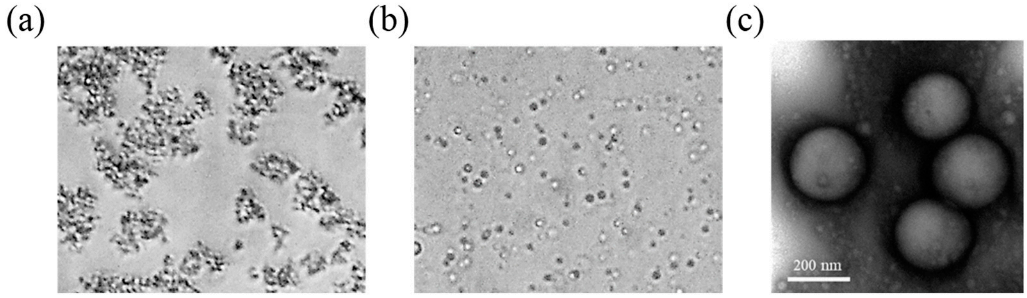

Figure S1. Morphology of (**a**) 3nAG suspension and (**b**) 3nAGN-NSC under 100X inverted microscope and (**c**) TEM image of 3nAGN-NSC.

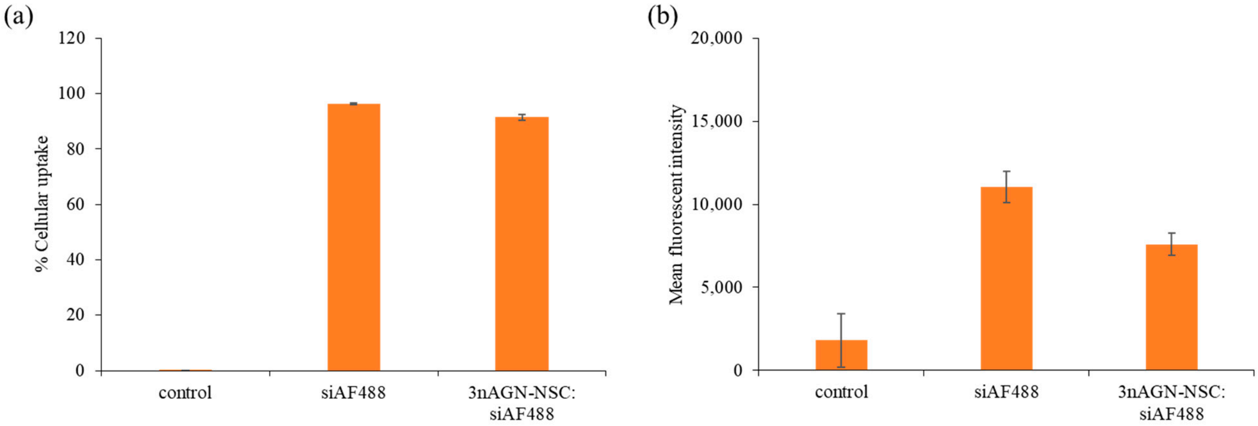

Figure S2. (**a**) Cellular uptake and (**b**) mean fluorescent intensity of siAF488 transfected with cationic niosomes and siAF488 in the combination with 3nAGN-NSC in MCF-7 cells.

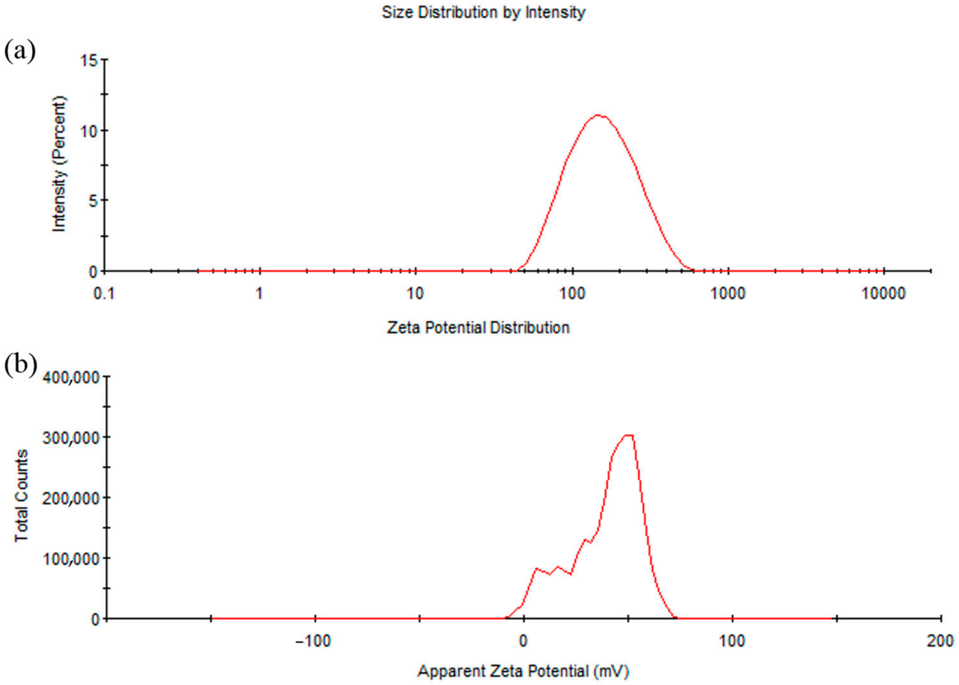

Figure S3. Characerization of cationic niosomes: (**a**) size distribution (mean particle size of 150.50 ± 5.52 nm) and (**b**) zeta potential distribution (mean zeta potential of +39.10 ± 1.89 mV) obtained from the Zetasizer Nano ZS.

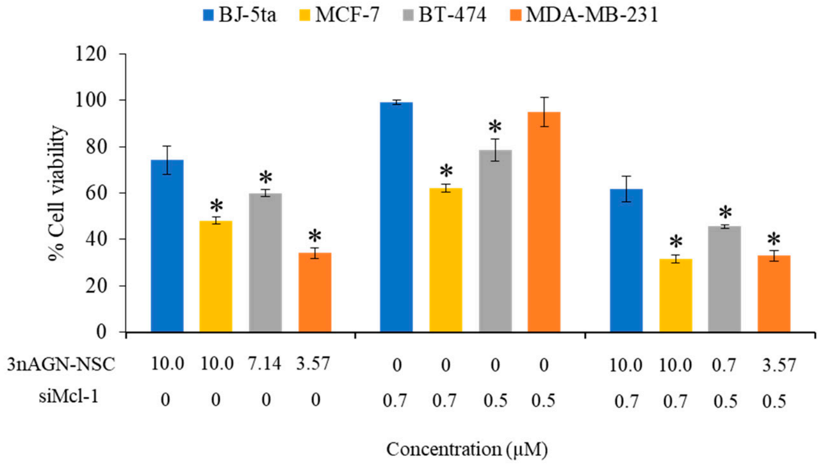

Figure S4. The cell viability of a single treatment with 3nAGN-NSC or siMcl-1 and the combination of 3nAGN-NSC: siMcl-1 (The tested concentration was nearly the IC50 of 3nAGN-NSC in each breast cancer cell) in normal human fibroblast cells (BJ-5ta cells), and breast cancer cells (MCF-7, BT-474 and MDA-MB-231 cells), which was examined by MTT assay. The cell was tested in the same condition with the method section 4.5. The BJ-5ta cells were maintained in DMEM containing 10% FBS, 1% L-glutamine, 1% non-essential amino acids solution, 100 U/ml penicillin, and 100 mg/ml streptomycin at 80% confluency before seeding. *The data is significantly different from BJ-5ta ($p < 0.05$).

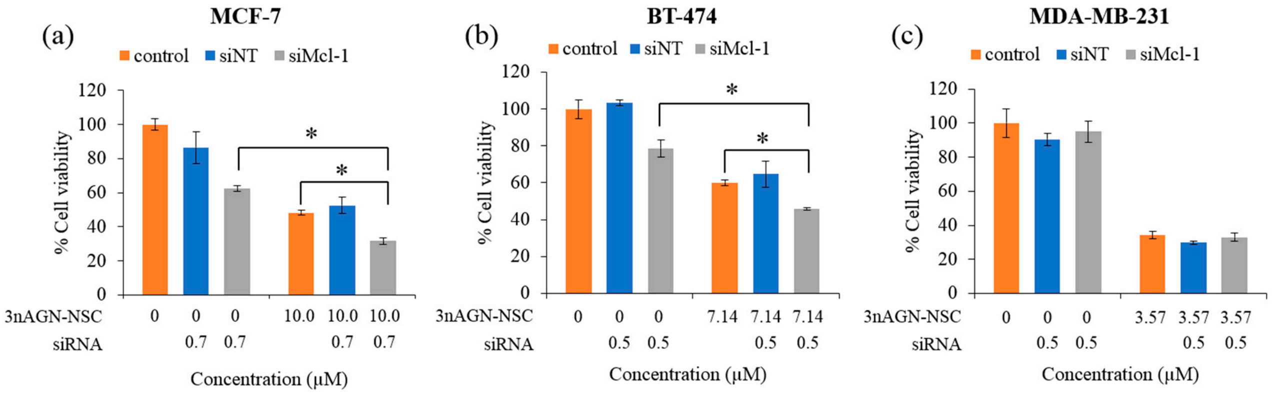

Figure S5. The cell viability of (**a**) MCF-7 with the combination 3nAGN-NSC: siMcl-1 (100: 0.07, 10 and 0.07 μM, respectively), (**b**) BT-474 after treatment with the combination 3nAGN-NSC: siMcl-1 (100: 0.07, 7.14 and 0.05 μM, respectively) and (**c**) MDA-MB-231 after treatment with the combination 3nAGN-NSC: siMcl-1 (50: 0.07, 3.57 and 0.05 μM, respectively). *The data is significantly different ($p < 0.05$).
